# Strict glycaemic control in patients hospitalised in a mixed medical and surgical intensive care unit: a randomised clinical trial

**DOI:** 10.1186/cc7017

**Published:** 2008-09-17

**Authors:** Gisela Del Carmen De La Rosa, Jorge Hernando Donado, Alvaro Humberto Restrepo, Alvaro Mauricio Quintero, Luis Gabriel González, Nora Elena Saldarriaga, Marisol Bedoya, Juan Manuel Toro, Jorge Byron Velásquez, Juan Carlos Valencia, Clara Maria Arango, Pablo Henrique Aleman, Esdras Martin Vasquez, Juan Carlos Chavarriaga, Andrés Yepes, William Pulido, Carlos Alberto Cadavid, Grupo de Investigacion en Cuidado intensivo: GICI-HPTU

**Affiliations:** 1 Department of Critical Care, Hospital Pablo Tobon Uribe, Calle 78B 69-240, Medellin, Colombia; 2 Department of Epidemiology, Hospital Pablo Tobon Uribe, Calle 78B 69-240, Medellin, Colombia; 3 Department of Internal Medicine, Universidad Pontificia Bolivariana, Cq 1 70-01, Medellin, Colombia; 4 Department of Internal Medicine, Hospital Pablo Tobon Uribe, Calle 78B 69-240, Medellin, Colombia; 5 Department of Internal Medicine, Universidad de Antioquia, Hospital Pablo Tobon Uribe, Calle 78B 69-240, Medellin, Colombia

## Abstract

**Introduction:**

Critically ill patients can develop hyperglycaemia even if they do not have diabetes. Intensive insulin therapy decreases morbidity and mortality rates in patients in a surgical intensive care unit (ICU) and decreases morbidity in patients in a medical ICU. The effect of this therapy on patients in a mixed medical/surgical ICU is unknown. Our goal was to assess whether the effect of intensive insulin therapy, compared with standard therapy, decreases morbidity and mortality in patients hospitalised in a mixed ICU.

**Methods:**

This is a prospective, randomised, non-blinded, single-centre clinical trial in a medical/surgical ICU. Patients were randomly assigned to receive either intensive insulin therapy to maintain glucose levels between 80 and 110 mg/dl (4.4 to 6.1 mmol/l) or standard insulin therapy to maintain glucose levels between 180 and 200 mg/dl (10 and 11.1 mmol/l). The primary end point was mortality at 28 days.

**Results:**

Over a period of 30 months, 504 patients were enrolled. The 28-day mortality rate was 32.4% (81 of 250) in the standard insulin therapy group and 36.6% (93 of 254) in the intensive insulin therapy group (Relative Risk [RR]: 1.1; 95% confidence interval [CI]: 0.85 to 1.42). The ICU mortality in the standard insulin therapy group was 31.2% (78 of 250) and 33.1% (84 of 254) in the intensive insulin therapy group (RR: 1.06; 95%CI: 0.82 to 1.36). There was no statistically significant reduction in the rate of ICU-acquired infections: 33.2% in the standard insulin therapy group compared with 27.17% in the intensive insulin therapy group (RR: 0.82; 95%CI: 0.63 to 1.07). The rate of hypoglycaemia (≤ 40 mg/dl) was 1.7% in the standard insulin therapy group and 8.5% in the intensive insulin therapy group (RR: 5.04; 95% CI: 1.20 to 21.12).

**Conclusions:**

IIT used to maintain glucose levels within normal limits did not reduce morbidity or mortality of patients admitted to a mixed medical/surgical ICU. Furthermore, this therapy increased the risk of hypoglycaemia.

**Trial Registration:**

clinicaltrials.gov Identifiers: 4374-04-13031; 094-2 in 000966421

## Introduction

Hyperglycaemia is frequently found in critically ill patients even in the absence of diabetes and it is associated with a poor prognosis [1-4]. A randomised trial of 1548 patients hospitalised in a surgical intensive care unit (ICU) showed that maintaining normal glucose levels reduces morbidity and mortality [5]. In another randomised study of 1200 patients requiring a minimum of three days hospitalisation in a medical ICU, intensive glucose control resulted in a decrease in morbidity but not in total mortality. However, a decrease in mortality was observed in a subgroup of patients treated with intensive control for three or more days [6].

Observational studies have suggested that strict glucose control is able to reduce hospital mortality in mixed medical/surgical ICUs [7,8], but other non-experimental studies in similar settings have not confirmed that the mean glucose level is an independent risk factor for ICU mortality. [9-11].

It remains unclear if intensive insulin therapy is equally efficacious in both medical and surgical patients [12]. Therefore, we conducted a randomised clinical trial to assess the efficacy and safety of intensive insulin therapy compared with standard glucose control in patients hospitalised for medical problems, surgical non-cardiovascular procedures or trauma in a mixed medical/surgical ICU.

## Materials and methods

### Patients

Patients aged 15 years or older admitted to the ICU at the Hospital Pablo Tobón Uribe (HPTU), Medellín, Colombia, between 12 July, 2003 and 21 December, 2005 with an expected ICU stay of at least two days were eligible for the trial. HPTU is a 239-bed university hospital with a mixed (surgical/medical) 12-bed adult ICU. Reasons for exclusion were pregnancy, diabetic ketoacidosis, hyperosmolar non-ketotic state, readmission to the ICU during the same hospitalisation, advanced stage cancer (solid or haematological), decision to withhold or withdraw aggressive therapies, and inclusion in another clinical trial.

The protocol was approved by the institution's ethics committee and written informed consent was obtained from the patients or their closest relatives. An independent Data Safety Monitoring Board comprised of three members with expertise in statistics, critical care and clinical epidemiology conducted two interim analyses. The end points for efficacy were based on the O'Brien-Flemming procedure with p values of 0.0006 and 0.0151. In both analyses they recommended to continue the trial.

### Randomisation

Patients were randomly assigned into study groups with a 1:1 ratio according to a computer-generated random number list with permuted blocks of six. They were stratified by diabetes diagnosis. The procedure was managed in the central pharmacy in charge of group assignment. Personnel involved in the treatment and investigation were unaware of the randomised schedule and the block size.

### Interventions

Patients were randomly assigned to receive either standard insulin therapy or intensive insulin therapy. Both groups received insulin via continuous infusion pump (Baxter colleague 3 or Baxter flo-Gard 6301, Baxter Healthcare Corporation I. V. System Division, Deerfield, IL, USA). The standard concentration of insulin (Humulin R, Eli Lilly and Company, Indianapolis, IN, USA) was 100 units in 100 ml of 0.9% saline solution. In the standard insulin group, insulin infusion was started when glucose levels exceeded 215 mg/dl and was adjusted to maintain blood glucose levels between 180 and 200 mg/dl (10.0 to 11.1 mmol/L) (See additional data file 1). In the intensive insulin group, insulin infusion was started when blood glucose levels exceeded 110 mg/dL, and was adjusted to maintain a glucose level of between 80 and 110 mg/dl (4.4 to 6.1 mmol/L) (See additional data file 2).

Blood glucose levels were measured in undiluted arterial blood. Undiluted samples were obtained by removing at least four times the flush-volume in the line between the sampling point and the arterial puncture site before the actual sample was taken or, when an arterial catheter was not available, in capillary blood with the use of a point-of-care glucometre (MediSense Optium, Abbot Laboratories MediSense Products Bedford, MA, USA). Glucose levels were determined with a glucometre at admission to ICU. They were repeated every one, two and four hours if the patient had insulin infusion, and every four and six hours if no insulin was required according to the algorithm.

A protocol (see additional data files 1 and 2), managed by the ICU nurses, was used for the adjustment of the insulin dose. The standard insulin therapy had been the usual treatment during the past 12 months, and a training period of three months in the intensive insulin therapy was implemented before starting the trial.

To prevent hypoglycaemia in patients who were receiving insulin but were not receiving enteral or total parenteral nutrition, 10% glucose was administered intravenously via continuous infusion (5 g/hour). The same infusion was used in patients with diabetes who were not receiving nutrition in order to prevent ketosis. It was also used for treatment of hypoglycaemic patients (glucose was administered via a 10 g intravenous boluses). The glucose infusion was stopped when the patient's nutrition was restarted or when the patient was no longer hypoglycaemic.

Protocols were consistently followed throughout the patient's whole ICU stay. After discharge from the ICU, treatment was continued according to the treating physician's recommendations and protocols were stopped.

We registered every patient's age, sex, body mass index, diabetes history, type of diabetes treatment, previous infections, comorbidities, ICU admitting diagnosis, Acute Physiology and Chronic Health Evaluation (APACHE II) score [13], Sequential Organ Failure Assessment (SOFA) score [14] and Glasgow coma score. The Glasgow coma score was obtained before starting sedation and was changed only when the sedation effects had finished.

Blood glucose levels were measured on admission. They were also measured daily in the mornings. The median of all daily values and daily maximal and minimal blood glucose levels were documented. Hypoglycaemic episodes of less than 41 mg/dl (2.2 mmol/l) and within 41 to 59 mg/dl (2.2 to 3.2 mmol/l) were registered, as well as the use of vasopressors, inotropics, steroids, transfusions, values of Glasgow trauma score, daily number of glucometre readings, creatinine levels and the SOFA scores.

If a patient presented with a temperature of 38.3°C or more or if the treating physician suspected an infection, blood, urine and sputum cultures were obtained. The diagnosis of infections acquired in the ICU was performed according to the CDC diagnosis criteria applied by three different physicians blinded to the treatment assignment [15]. A distinction was made between primary and secondary bacteraemia, depending on whether or not a focus could be identified.

### Outcomes

The primary outcome was 28-day all-cause mortality. Secondary outcomes were: ICU mortality; hospital mortality; incidence of infections in the ICU (ventilator-associated pneumonia, urinary infections, catheter-related infections and primary bacteraemias); ICU length of stay; days of mechanical ventilation and incidence of severe hypoglycaemia defined as number of patients with at least one episode of blood glucose level less than 40 mg/dl (2.2 mmol/l).

### Sample size

We estimated that the enrollment of 504 patients would provide a power of 80% to detect an absolute reduction of 10% in the 28-day mortality rate with an alpha error (two-sided test) of 0.05. We assumed a 25% mortality rate in the control group.

### Statistical analysis

Data is presented in absolute numbers and proportions for nominal variables. Mean ± standard deviation (SD) or median and interquartile range (IQR) is used for continuous variables, normally or non-normally distributed, respectively.

The outcomes were analysed according to the intention-to-treat principle. Primary and secondary end points were compared with the use of a Student's t-test for parametric data, the Mann-Whitney U test for non-parametric data, and the Pearson chi-square or Fisher exact test for proportions. For rates of mortality, 95% confidence intervals (CI) were calculated, and a p < 0.05 was considered statistically significant. No corrections were made for multiples tests. The statistical analyses were executed with the statistics packet SPSS/PC 13.0 (SPSS Inc., Chicago, IL, USA).

## Results

**Figure 1:**
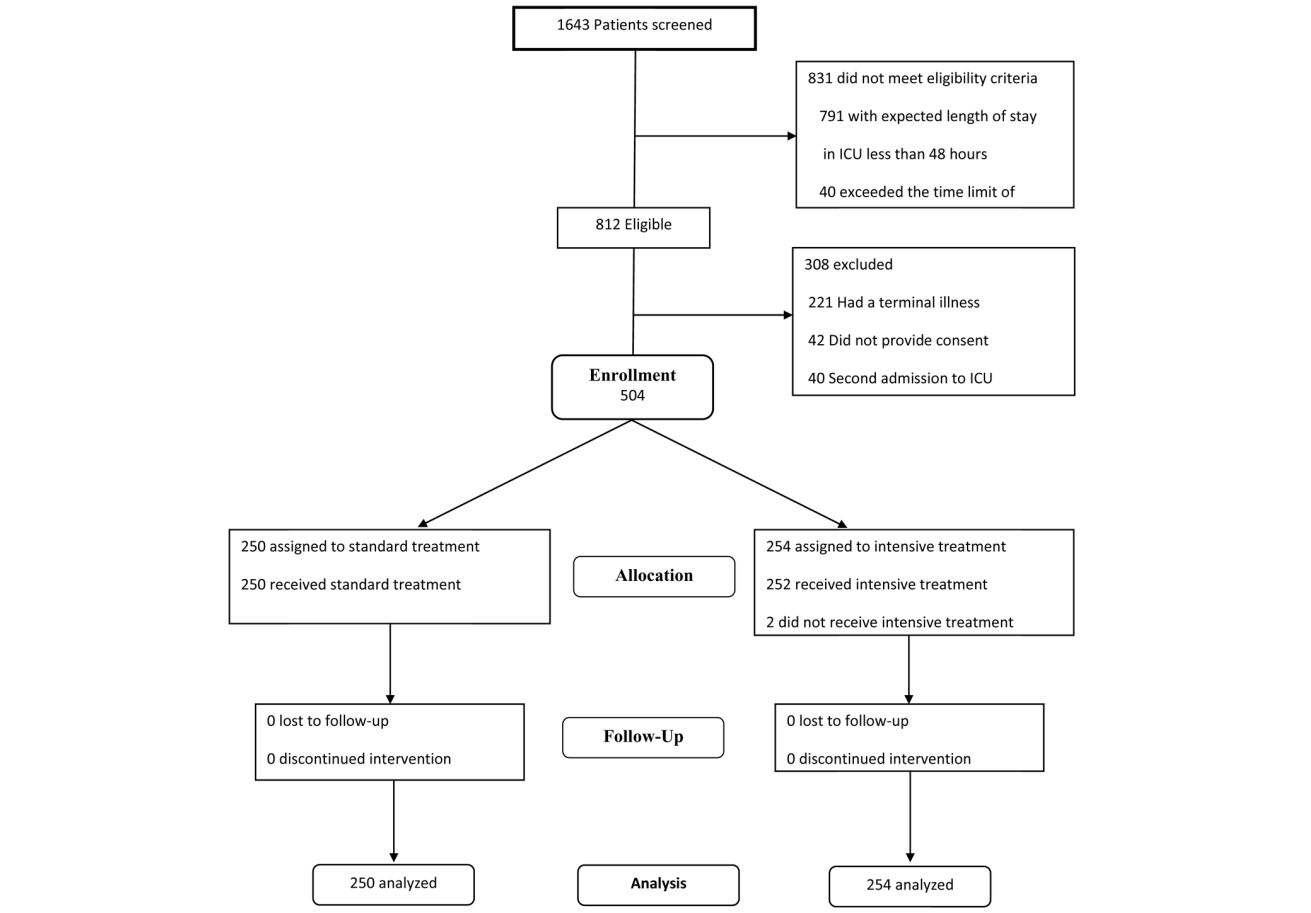
Flow of participants through the trial.

During the study period 1643 patients were admitted to the ICU and 831 did not meet inclusion criteria: 791 had an expected length of stay in the ICU of less than 48 hours and 40 exceeded the recruitment time limit. Of the 812 patients who met the inclusion criteria, 308 were excluded for the following reasons: 221 had a terminal illness, 42 refused to participate, 40 had a second admission to the ICU and five had diabetic ketoacidosis or hyperosmolar coma. A total of 504 patients were enrolled, 250 in the control group and 254 in the intervention group. There was one patient from the intensive insulin group who did not receive either of the two protocols and one patient who belonged to the intensive insulin group who received the conventional insulin protocol. According to the intention-to-treat principle, they were analysed in the group they had been assigned to originally. The patients were followed-up until discharged from the hospital (Figure 1).

Demographics and baseline characteristics were similar in the two groups (Table 1). The average delay between admission to the ICU and enrollment into a protocol group was 12.5 ± 6.2 hours in the intensive insulin group and 12.4 ± 5.9 hours in the standard insulin group (p = 0.853). The mean time required to reach the glucose goal was 6.3 ± 2.1 hours in the intensive insulin group and 6.1 ± 2.5 hours in the standard insulin group (p = 0.332).

**Table 1:** Baseline characteristics of the patients.

**Variable**	**Standard treatment**	**Intensive treatment**
	(N = 250)	(N = 254)
Male sex (%)	154 (62)	147 (58)
Age (years)*	47.4 ± 19.3	45.9 ± 20.2
Body-mass index *†	25 ± 4.5	24.6 ± 4,2
History of diabetes (%)	29 (11.6)	32 (12.6)
Treated with insulin	9 (3.6)	5 (2.1)
Histrory cirrhosis	7 (2.8)	9 (3.5)
History heart failure	3 (1.2)	6 (2.4)
History kidney failure	16 (6.4)	10 (3.9)
History of cancer	9 (3.6)	15 (5.9)
APACHE II – score *‡	15.6 ± 7.6	15.7 ± 6.9
SOFA – scores *§	7.6 ± 3.5	7.3 ± 3.2
Reason for ICU admission (%)		
Medical	123 (49.2)	123 (48.4)
Surgery	37 (14.8)	45 (17.7)
Trauma	90 (36)	86 (33.9)
Blood glucose on admission (mg/dl) *¶	153.6 ± 67.1	155.3 ± 68.4

* Values presented as mean ± SD.

†The body mass index is the weight in kilograms divided by the square of the height in metres.

‡APACHE II = Acute Physiology and Chronic Health Evaluation. Higher scores reflects more severe critical illness.

§SOFA = Sequencial Organ Failure Assessment. Higher scores reflect more severe organic dysfunction. for the worst values in the six organs during the first 24 hours after enrollment.

¶To convert the values for glucose to millimoles per litre, multiply by 0.05551.

Admissions due to infections were similar in both groups: 82 patients of 250 (32.8%) in the standard insulin group and 83 of 254 (32.7%) in the intensive insulin group.

**Figure 2:**
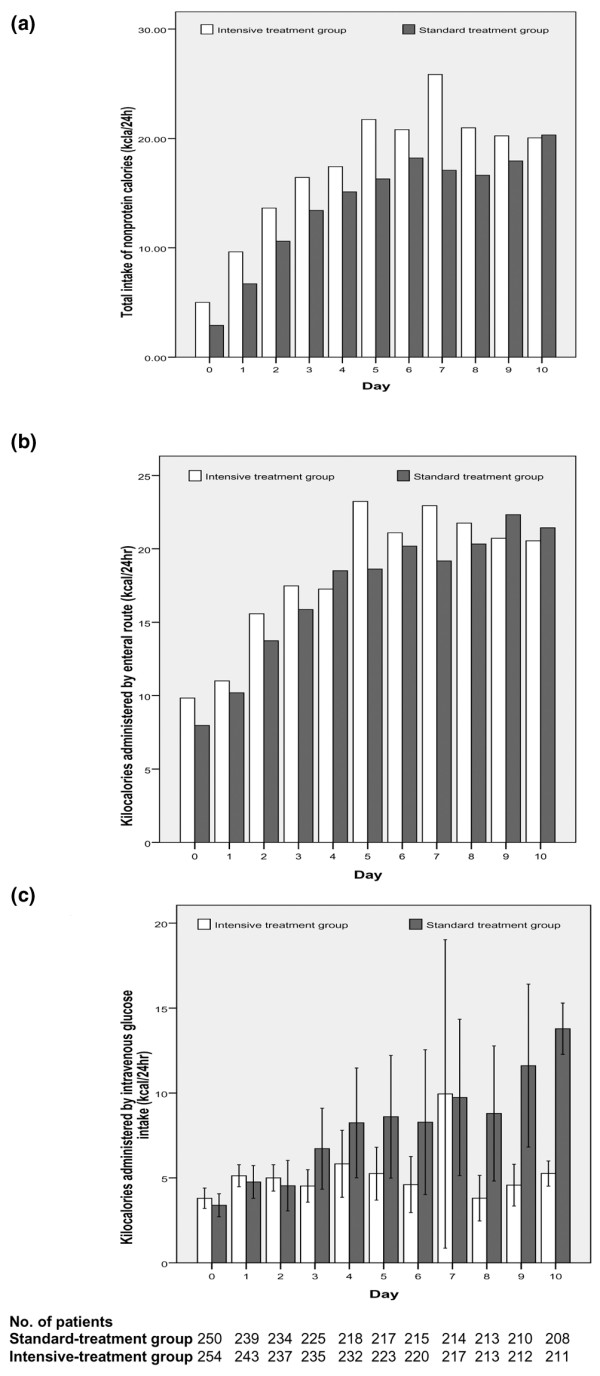
**Nutrition administered to all 504 patients during the first 10 days of intensive care**. Feeding at 0 represents the administration of nutrition between admission and 7 a.m., and 1 represents feeding on the first day after admission, from 7 a.m. onwards. Nutrition in the two groups was similar. (a) Total caloric intakes areexpressed as mean values (with the 95% confidence intervals indicated by the error bar). (b) Nutrition administered by the enteral route are expressed as mean values, (with the 95% confidence intervals indicated by the error bar). (c) Nutrition administered by the parenteral route are expressed as mean values (with the 95% confidence intervals indicated by the error bar).

The mean calorie intake in 24 hours was 23.1 ± 12.7 kcal/kg in the standard insulin group and 25.5 ± 14.4 kcal/kg in the intensive insulin group (mean difference: 2.4; 95% CI: -0.02 to 4.9). Total parenteral nutrition (glucose 30 to 50% plus amino acids and lipids to reach the required total caloric intake) alone or combined with enteral nutrition was given to 14 patients in the standard insulin group (5.6%) and 14 in the intensive insulin group (5.5%). The remaining patients received total-enteral feeding exclusively. In the standard insulin group 47% patients (118 of 250) received at least six hours intravenous 10% glucose (5 g/hour) during the ICU stay (Figure 2).

**Figure 3:**
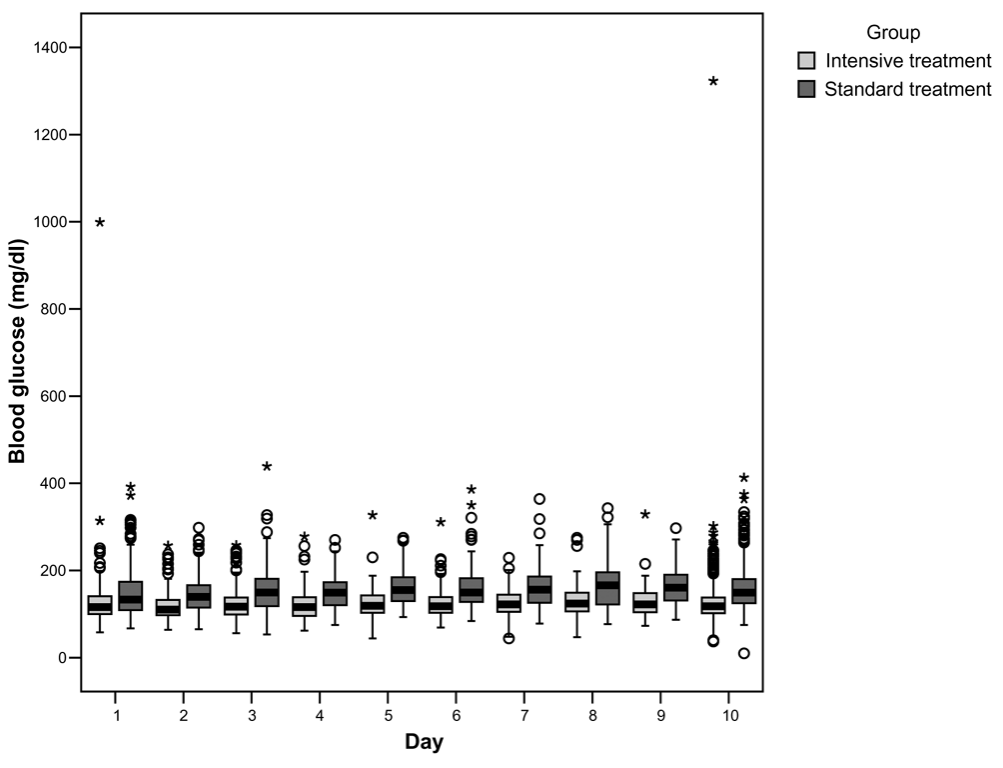
**Daily blood glucose levels during the first 10 days of intensive care**. Medians and interquartile ranges (IQR) during the ICU stay (time) are shown for the two treatment arms.

More patients in the intensive insulin group than in the standard insulin group received insulin (97% vs. 47%, p < 0.001) as well as having a higher amount of insulin administered per 24 hours (52.4 ± 53.3 IU vs. 12.5 ± 32.8 IU, p < 0.001). The intensive insulin group had lower mean blood glucose level than the standard insulin group: 117 mg/dl (IQR: 101 to 140) compared with 148 mg/dl (IQR: 122 to 180), (p < 0.001) (Figure 3), and had more glucometre readings per day: 13 ± 5.5 compared with 5.9 ± 4.0, p < 0.001. The proportion of patients with at least one episode of a glucose level of 40 mg/dl or less was higher in the intensive insulin group (8.3% vs. 0.8%, p < 0,001). Six patients in the intensive insulin group had two or more hypoglycaemic events (Table 2). One patient presented with an episode of tonic-clonic generalised seizure associated with hypoglycaemia. The seizure was controlled with insulin suspension and the administration of 200 cc 10% glucose bolus with good response and no neurological damage.

**Table 2:** Insulin therapy and control of blood glucose levels.

**Variable**	**Standard treatment **(N = 250)	**Intensive treatment **(N = 254)	**P value †**
Administration of insulin (%)	118 (47)	246 (97)	< 0.001
Insulin dose (IU/day) *	12.5 ± 32.8	52.4 ± 53.3	< 0.001
Morning blood glucose (mg/dl) ‡ – Median. (Interquartile range)	148 122 to 180	117 101 to 140	< 0.001
Minimal blood glucose (mg/dl) – Median. (Interquartile range)	122 105 to 143	82 72 to 94	< 0.001
Maximal blood glucose (mg/dl) – Median. (Interquartile range)	172 141 to 215	162 140 to 193	< 0.001
Median blood glucose (mg/dl) – Median. (Interquartile range)	149 124.5 to 180	120 109.5 to 134	< 0.001
Number of blood glucose measurement per day *	5.9 ± 4	13 ± 5.5	< 0.001
Number of patients in which morning median blood glucose was in their preset range (%)	43 (17.2)	100 (39.4)	< 0.001
Hypoglycaemia ≤ 40 mg/dl (%)	2 (0.8)	21 (8.3)	< 0.001

* Values presented as mean ± SD. Hypoglycaemia are number of patients with at least one episode over total number of patients per group.

† P values were determined wit the use of Student's t-test, Mann-Whitney test or Chi-square test as appropriate.

‡ To convert the values for glucose to millimoles per litre, multiply by 0.05551.

The median length of stay in the ICU, the duration of mechanical ventilation and the rate of ICU-acquired infections were not reduced by intensive insulin therapy. The use of medications other than insulin was the same for both groups. No differences were found in new onset acute renal failure, requirement of haemodialysis or red blood cell transfusions (Table 3).

**Table 3:** Causes of morbidity in the patient group

Variable	**Standard treatment (n = 250)**	**Intensive treatment (n = 254)**	P value†
Length of stay in ICU (days)			
Median (Interquartile range)	6 (3 to 11)	6 (3 to 12)	0.351
Duration of ventilatory support (days)			
Median (Interquartile range)	5 (2 to 9)	6 (2 to 10)	0.857
Ventilatory support (%)	238 (95.2)	238 (93.7)	0.476
Dopamine (%)	190 (76)	182 (71.7)	
Dosage (μg/kg/minute) *‡	14.45 ± 8.97	14.85 ± 8.99	0.366
Norepinephrine (%)	68 (27.2)	55 (21.7)	
Dosages (μg/kg/minute) *‡	0.36 ± 0.77	0.36 ± 0.56	0.993
Epinephrine (%)	44 (17.6)	46 (18.1)	
Dosages (μg/kg/minute) *‡	0.34 ± 0.40	0.31 ± 0.59	0.613
Dobutamine (%)	9 (3.6)	14 (5.5)	
Dosages (μg/kg/minute) *‡	4.97 ± 2.43	8.43 ± 6.48	0.060
Swan Ganz (%)	28 (11.2)	30 (11.8)	0.800
Renal impairment (%) §	25 (10)	32 (12.6)	0.357
Renal-replacement therapy (%)	33 (13)	27 (10.8)	0.447
Hydrocortisone (%)	66 (26.4)	67 (26.3)	0.996
Ventilator associated pneumonia (%)	55 (22)	43 (16.9)	0.15
Catheter-related infection (%)	9 (3.6)	6 (2.4)	0.414
Urinary infections (%)	11 (4.4)	13 (5.1)	0.705
Primary bacteraemia (%)	8 (3.2)	7 (2.8)	0.769
Treatment with antibiotics for ≥ 8 days (%)	183 (73.2)	186(73.2)	0.994
Red-cell transfusions			
Patients requiring transfusions (%)	91 (36)	99 (39)	0.551
No. of transfusions/patients*	6 ± 5.5	4.9 ± 4.1	0.412

* Values presented as mean ± SD.

† P values were determined wit the use of Student's t-test, the chi-square test or Fischer test as appropriate.

‡ Maximal dosage per day

§Renal impairment: peak plasma creatinine > 2.5 mg/dl, Peak plasma urea nitrogen > 60 mg/dl, dialysis or continuous venovenous haemofiltration.

All-cause mortality at 28 days was 32.4% (81 of 250) in the standard insulin group and 36.6% (93 of 254) in the intensive insulin group. ICU mortality was similar for patients of the standard insulin group and in those from the intensive insulin group: 78 of 250 patients (31.2%) and 84 of 254 (33.1%), respectively. Hospital mortality was also similar between the standard insulin group and the intensive insulin group: 96 of 250 (38.4%) and 102 of 254 (40.2%), respectively (Table 4).

**Table 4:** Causes of mortality in the patient group

**Variable**	**Standard treatment (N = 250)**	**Intensive treatment (N = 254)**	**Relative risk (95% confidence interval)**
28-day deaths	81 (32.4)	93 (36.6)	1.1 (0.85 to 1.42)
Death in the intensive care unit (%)	78 (31.2)	84 (33.1)	1.06 (0.82 to 1.37)
In-hospital death (%).	96 (38.4)	102 (40)	1.05 (0.84 to 1.3
Death with history of diabetes	9 of 29 (31)	12 of 32 (37.5)	1.21 (0.60 to 2.40)

## Discussion

We found that intensive glucose control did not reduce the morbidity or the mortality of patients admitted to a mixed medical/surgical ICU with medical problems, non-cardiovascular surgeries or trauma. These results differ from two previous studies. The first one with patients in a cardiovascular-surgical ICU [5] demonstrated a decrease in morbidity and mortality. The other in patients in a medical ICU demonstrated a decrease in morbidity; however, a decrease in mortality was only seen in a subgroup of patients with an ICU stay longer than two days [6].

A possible explanation for these differences could be the different type of patients in each study. The first study was conducted in a surgical ICU where 63% of the patients had cardiovascular problems. In these patients, the decrease in mortality recorded for the intensive insulin group was associated with a decrease in both the frequency of infections (46%) and in the number of deaths due to multiple organ failure of known sepsis origin [5]. In contrast, our study was conducted in a mixed medical/surgical ICU where the patients were admitted with medical problems, non-cardiovascular surgeries or trauma, and where established infection was a common reason for admission (33%). In addition, the intervention did not significantly decrease the rate of ICU-acquired infections (33.2% in the intensive insulin group compared with 27.17% in the standard insulin group). These findings suggest that prevention of nosocomial infections, more than control of established ones, could be a major mechanism for the mortality reduction in patients treated with strict glucose control. Furthermore, the recently finished Volume Substitution and Insulin Therapy in Severe Sepsis trial, a randomised multicentre trial designed to assess the efficacy and safety of intensive insulin therapy in patients with severe sepsis and septic shock, was stopped early for safety reasons [16]. Of the 537 evaluated patients there was no significant difference between the two groups in the 28-day mortality rate or the mean organ failure score. The rate of severe hypoglycaemia, however, was higher in the intensive insulin therapy group compared with the standard insulin therapy group (17.0% vs. 4.1%, p < 0.001).

The patients in our study were younger (47 years old) than in other studies (63 years old) [5,6], and on admission to the ICU the mean APACHE II score was lower in our study compared with the medical ICU study by Van den Berghe and colleagues (15 vs. 23) [6]. In addition, our population was relatively healthy before the acute process that indicated ICU admission, as less than 14% of them had a significant concomitant disease before admission. Thus, our study population may not be critically ill enough to obtain a benefit from intensive insulin therapy.

The patients in the intensive group in our study did not reach the normal glucose level because our protocols were carefully designed to avoid a high rate of hypoglycaemia. Therefore, this strict control against hypoglycaemia could also become a measure favouring the balance in glucose goals between the groups. Furthermore, the mean values for glucose level in the standard group were lower than expected because our patients did not routinely receive a 10% dextrose infusion, and a lower amount of parenteral calories was supplied from the beginning. Thus, the median difference in glucose values between groups was about 30 mg/dl and although this difference was statistically significant, there was a considerable overlap between the two study groups (Figure 3). Such a relatively small effect over glucose control could be one of the reasons no differences were seen in morbidity or mortality rates.

In addition, we observed a large variability of blood glucose concentration in both groups, which has been suggested as another possible explanation for the lack of beneficial effects of insulin therapy [17]. The delay in the recruitment, much longer than the studies by Van den Berghe and colleagues [5,6], may explain our findings as it is possible that any benefit may only be accrued early on.

Severe hypoglycaemia of 40 mg/dl or less was associated with the application of insulin in our setting, as well as in the cardiovascular surgical ICU study [5], but less frequent than in the medical ICU study [6]. Hypoglycaemia of 60 mg/dl or less was also more frequently associated with the utilisation of insulin in the intensive group 66% compared with 10% in the conventional group.

There were some limitations in our research. These were related to sample size, which was underpowered to detect both overall differences and those within subgroups. At the time when we planned and conducted our study the only available information about efficacy was inferred from the first trial by Van Den Berghe and colleagues [5], which showed a 42.5% relative risk reduction over a mortality rate in the control group of about 8%. Based on these data, we assumed the same relative risk reduction but over a higher expected mortality in the control group (i.e. 25%). Therefore, our study is not large enough to say that there was no benefit in the overall population or in the subgroups of medical or trauma/surgery patients. On the other hand, the inability to maintain the blinding because the titration of insulin required monitoring of glucose levels may be a potential source of bias. In order to decrease this problem, those physicians evaluating ICU-acquired infections were blinded to the study group. Finally, this study was performed in only one centre, an obvious constraint to generalise our results.

## Conclusion

We found that strict glucose control did not decrease morbidity or mortality in patients hospitalised in a mixed medical/surgical ICU. Instead, the intervention produced an important increase in severe hypoglycaemia. Of note, however, was that it was very difficult to strictly restrict glycaemic control and the study showed that less than 50% of patients were within target range. Therefore, the combination of an insufficient difference between the treatment groups in blood glucose values and lack of power makes it impossible to draw any conclusion on the efficacy of tight glycaemic control. Multicentre studies are required to confirm these findings.

## Key messages

• It was very difficult to tightly control glycaemic levels in patients hospitalised in a mixed medical/surgical ICU and less than 50% of the patients were within target range.

• In this patient population with medical problems, non-cardiovascular surgeries and trauma, intensive insulin therapy did not reduce the mortality or morbidity of patients admitted to a mixed medical/surgical ICU.

• Intensive insulin therapy was associated with an increased risk of severe hypoglycaemia.

• Larger multicentre clinical trials are required to confirm these findings

## Abbreviations

APACHE II: Acute Physiology and Chronic Health Evaluation; CDC: Centers for Disease Control; 95% CI: 95% confidence interval; HPTU: Hospital Pablo Tobón Uribe; ICU: intensive care unit; IQR: interquartile range; RR: relative risk; SD: standard deviation; SOFA: Sequential Organ Failure Assessment.

## Competing interests

The authors declare that they have no competing interests.

## Authors' contributions

GD, JD, AR, AQ and LG participated in study conception, study design, data acquisition, data analysis and interpretation, and drafting of the manuscript, NS, MB, JT, JV, JV, CA, PA, EV, JCH, AY, WP and CC participated in the study design, data acquisition and drafting of the manuscript. All authors read and approved the final manuscript.

## Supplementary Material

Additional file 1 a word file containing text that describes the standard insulin therapy protocol. Click here for file

Additional file 2 a word file containing a text that describes the intensive therapy protocol. Click here for file
