# Supplementary material for: Strict glycaemic control in patients hospitalised in a mixed medical and surgical intensive care unit: a randomised clinical trial
Source: Crit Care. 2008 Sep 17;12(5):R120. doi: 10.1186/cc7017 (PMC2592751; doi:10.1186/cc7017)
Supplement: Additional file 1 — a word file containing text that describes the standard insulin therapy protocol. [file cc7017-S1.doc]

# Standard insulin therapy protocol

# Guidelines for insulin titration in the ICU

1. **Insulin infusion**

Concentration and mode of administration: Insulin is only given by continuous intravenous infusion. The standard concentration is 100 IU insulin in 100mL NaCl-0.9%.

1. **Measurement of blood glucose levels**

Whole blood glucose levels were measured in undiluted arterial blood. Undiluted samples are obtained by removing at least 4 times the flush-volume in the line between the sampling point and the arterial puncture site before the actual sample is taken or, when an arterial catheter was not available, in capillary blood, with the use of a point-of-care glucometer.

After admission to ICU and until gold (180-200 mg/dL or 10.0 - 11.1 mmol/L) is reached, hourly or two-hourly measurement of blood glucose is advised, and repeated every 1, 2 and 4 hours if the patient had insulin infusion and every 4 and 6 hours if no insulin was required. Thereafter, every 4 hours measurement of blood glucose suffices. In case of hypoglycemia or steep falls or rises in glycemia, more frequent control than every 1h is advised.

1. **Titration schedule**

*3.1. Starting up insulin infusion and initial stabilization of blood glucose level*

- When blood glucose level was 216 – 249 mg/dL insulin infusion was initiated at a starting dose of 1 IU/hr. When blood glucose level on which insulin is started is > 250 mg/dL, the starting dose of insulin can be set at 2 IU/hr.
- When a control blood glucose level was > 250 mg/dL, the insulin dose was increased by increments of 2 IU/hr.
- When control blood glucose level was 215-249 mg/dL, the insulin dose was increased by increments of 1 IU/hr.
- Once the blood glucose level was between 180 and 200 mg/dL, the insulin dose was kept constant.
- When the blood glucose level decreased to <180 mg/dL, insulin infusion was decreased until the blood glucose level was between 180 and 200 mg/dL. The insulin dose was further decreased and eventually completely stopped when blood glucose levels decreased further. Insulin infusion was only re-started when blood glucose exceeded 215 mg/dL.
  1. *Dose adjustments after initial stabilization*
- When blood glucose was 150-179 mg/dL, the dose was reduced to half if insulin infusion was > 2 IU/hr and stopped if insulin infusion was ≤ 2 IU/hr. Then blood glucose level was checked within the next hour.
- When blood glucose was -149 mg/dL, insulin infusion was stopped and the blood glucose level was checked within the next hour.
- When blood glucose was < 60 mg/dL, insulin infusion was stopped, adequate baseline glucose intake was assured, glucose was administered via 10- g intravenous boluses and the blood glucose level was checked within the next hour.
- After the patients discharge from the ICU, the protocol was stopped.

1. **Special concerns regarding alterations in caloric intake**

Adequate caloric and glucose intake is essential.

At the time of interruptions of tube feeding, the insulin infusion was stopped in order to avoid hypoglycemia.

At the time of patient transportation to an investigation or to the operating room for surgery, all IV and enteral administration of feeding is usually stopped at that time and insulin infusion should also be stopped. Check blood glucose level after having done so and ensure an adequate level before transport.

Whenever a patient is extubated and assumed to re-start normal oral food intake, the IV or tube feeding is usually reduced in order to allow appetite to re-occur. In those patients it is crucial to reduce the insulin dose proportionately (often temporarily stopped).
