# Supplementary material for: Strict glycaemic control in patients hospitalised in a mixed medical and surgical intensive care unit: a randomised clinical trial
Source: Crit Care. 2008 Sep 17;12(5):R120. doi: 10.1186/cc7017 (PMC2592751; doi:10.1186/cc7017)
Supplement: Additional file 2 — a word file containing a text that describes the intensive therapy protocol. [file cc7017-S2.doc]

# Intensive insulin therapy protocol

1. **Insulin infusion**

Concentration and mode of administration: Insulin is only given by continuous intravenous infusion. The standard concentration is 100 IU insulin in 100mL NaCl-0.9%.

1. **Measurement of blood glucose levels**

Whole - blood glucose levels are measured in undiluted arterial blood. Undiluted samples are obtained by removing at least 4 times the flush-volume in the line between the sampling point and the arterial puncture site before the actual sample is taken or, when an arterial catheter was not available, in capillary blood, with the use of a point-of-care glucometer.

After admission to ICU and until normoglycemia (80-110 mg/dL or 4.4-6.1 mmol/L) is reached, hourly or two-hourly measurement of blood glucose is advised, and repeated every 1, 2 and 4 hours if the patient had insulin infusion and every 4 and 6 hours if no insulin was required. In case of hypoglycemia or steep falls or rises in glycemia, more frequent control than every 1 h is advised.

1. **Titration schedule**

*3.1. Starting up insulin infusion and initial stabilization of blood glucose level*

- When blood glucose level was 111 – 125 mg/dL, insulin was started at 1 IU/hr. (1.5 IU/hr if the first blood glucose level was 126 - 199 mg/dL and 2 IU if the first blood glucose level exceeded 200 mg/dL).
- When te next blood glucose level exceeded 250 mg/dL, insulin was increased by 2 IU/hr.
- When the next blood glucose level was 181 - 249 mg/dL, insulin was increased 1.5 IU/h.
- When the next blood glucose level was 151 - 180 mg/dL, insulin was increased 1 IU/h.
- When the next blood glucose level was 131 - 150 mg/dL, insulin was increased 0.5 IU/h.
- When the next blood glucose level was 110-130 mg/dL, insulin was adjusted by 0.1 to 0.4 IU/hr.
- When blood glucose level was 80–110 mg/dL, insulin dose was unaltered.
  1. *Dose adjustments after initial stabilization*
- When blood glucose decreased by more than 50% change, the dose was reduced to half and the blood glucose level was checked within the next hour.
- When blood glucose was 111 – 130 mg/dL, adjust with increments/decrements of 0.1 –0.4 IU per hour (111 - 115: 0.1 IU/h, 116 - 120: 0.2 IU/h, 121 - 125: 0.3 IU/h, 126 - 130: 0.4 IU/h).
- When blood glucose level was 80-90 mg/dL, if level falls more than 20 mg/dL after a dose adjustment, reduce the insulin dose to half and check blood glucose level within the next hour.
- When blood glucose level was 70-79 mg/dL, reduce the insulin dose to half if insulin infusion was > 1 unit per hour and stop if insulin infusion was ≤ 1 unit per hour and check the blood glucose level within the next.
- When blood glucose was 60-69 mg/dL, insulin infusion was stopped and check the blood glucose level within the next hour.
- When blood glucose was < 60 mg/dL, insulin infusion was stopped, adequate baseline glucose intake was assured, glucose was administered via 10- g intravenous boluses and the blood glucose level was checked within the next hour .
- After the patients discharge from the ICU, the protocol was stopped.

1. **Special concerns regarding alterations in caloric intake**

Adequate caloric and glucose intake is essential.

At the time of interruptions of tube feeding, the insulin infusion was stopped in order to avoid hypoglycemia.

At the time of patient transportation to an investigation or to the operating room for surgery, all IV and enteral administration of feeding is usually stopped at that time and insulin infusion should also be stopped. Check blood glucose level after having done so and ensure an adequate level before transport.

Whenever a patient is extubated and assumed to re-start normal oral food intake, the IV or tube feeding is usually reduced in order to allow appetite to re-occur. In those patients it is crucial to reduce the insulin dose proportionately (often temporarily stopped).
